# Prosociality, Cognition, & Health: Diversity and Consistency of Prosocial Behaviors Matters

**DOI:** 10.1093/geroni/igaf122.2567

**Published:** 2025-12-31

**Authors:** Morgan Taylor, Margie Lachman

**Affiliations:** Brandeis University, Waltham, Massachusetts, United States; Brandeis University, Waltham, Massachusetts, United States

## Abstract

Research demonstrates that being prosocial—engaging in behaviors that benefit others—relates to better cognition and health outcomes across the lifespan. However, the effect of engaging in multiple prosocial behaviors consistently over time has yet to be investigated. Using the Midlife in the United States (MIDUS) study, we examined the relationship between three prosocial behaviors (volunteering, informally helping others, donating money), cognition (episodic memory, executive function), and functional health at Wave 3 (2013/2014). Participants (N = 2,096, ages 42-92) reported how many hours/month they volunteered and informally helped others and dollars/month they donated to organizations. We created a categorical variable for each behavior wherein participants received 0 if they didn’t engage in those behaviors and 1 if they did. Replicating past work, we found those who engaged in each prosocial behavior had better cognition and functional health than those that did not, controlling for age, sex, education, race, and income. Critically, in a separate model, we found age moderated the effect of informal helping on executive function; informally helping others was more beneficial for older adults than younger adults. Furthermore, we found participants who engaged in more prosocial behaviors (2 or 3) had better cognition and health than those that engaged in fewer (0 or 1). Finally, we found those who consistently volunteered and donated over 10 years had better cognition than those who did so inconsistently. These results suggest that consistently participating in a variety of prosocial behaviors could be protective for middle and older adults’ cognition and health.

